# Long-Term Chronic Intermittent Hypobaric Hypoxia in Rats Causes an Imbalance in the Asymmetric Dimethylarginine/Nitric Oxide Pathway and ROS Activity: A Possible Synergistic Mechanism for Altitude Pulmonary Hypertension?

**DOI:** 10.1155/2016/6578578

**Published:** 2016-05-30

**Authors:** Nicole Lüneburg, Patricia Siques, Julio Brito, Karem Arriaza, Eduardo Pena, Hans Klose, Fabiola Leon-Velarde, Rainer H. Böger

**Affiliations:** ^1^Department of Clinical Pharmacology and Toxicology, University Medical Center Hamburg-Eppendorf, 20246 Hamburg, Germany; ^2^Institute of Health Studies, Arturo Prat University, Iquique, Chile; ^3^Department of Pneumology, University Medical Center Hamburg-Eppendorf, 20246 Hamburg, Germany; ^4^Department of Biological and Physiological Sciences, Faculty of Sciences and Philosophy/IIA, Cayetano Heredia University, Lima, Peru

## Abstract

Chronic intermittent hypoxia (CIH) and chronic hypoxia (CH) are associated with high-altitude pulmonary hypertension (HAPH). Asymmetric dimethylarginine (ADMA), a NO synthase (NOS) inhibitor, may contribute to HAPH. This study assessed changes in the ADMA/NO pathway and the underlying mechanisms in rat lungs following exposure to CIH or CH simulated in a hypobaric chamber at 428 Torr. Twenty-four adult Wistar rats were randomly assigned to three groups: CIH2x2 (2 days of hypoxia/2 days of normoxia), CH, and NX (permanent normoxia), for 30 days. All analyses were performed in whole lung tissue. L-Arginine and ADMA were analyzed using LC-MS/MS. Under both hypoxic conditions right ventricular hypertrophy was observed (*p* < 0.01) and endothelial NOS mRNA increased (*p* < 0.001), but the phosphorylated/nonphosphorylated vasodilator-stimulated phosphoprotein (VASP) ratio was unchanged. ADMA increased (*p* < 0.001), whereas dimethylarginine dimethylaminohydrolase (DDAH) activity decreased only under CH (*p* < 0.05). Although arginase activity increased (*p* < 0.001) and L-arginine exhibited no changes, the L-arginine/ADMA ratio decreased significantly (*p* < 0.001). Moreover, NOX4 expression increased only under CH (*p* < 0.01), but malondialdehyde (MDA) increased (up to 2-fold) equally in CIH2x2 and CH (*p* < 0.001). Our results suggest that ADMA and oxidative stress likely reduce NO bioavailability under altitude hypoxia, which implies greater pulmonary vascular reactivity and tone, despite the more subdued effects observed under CIH.

## 1. Background

Working at high altitude and resting at sea level for many years expose humans to an unusual labor-related condition called long-term chronic intermittent hypobaric hypoxia (CIH) [1]. Exposure to high altitude causes reduced arterial oxygen saturation that, in turn, may elicit various pathophysiological sequelae depending on whether this exposure is acute or chronic. Both acute and chronic exposure cause pulmonary arterial hypertension and an increase in blood hemoglobin levels [2]. Thus, hypobaric hypoxia-induced pulmonary arterial hypertension (HAPH) is a relevant problem that affects populations living and working at high altitudes, such as those in the Andean region and on the Himalayan plateau, with a prevalence varying between 10 and 15% [3].

CIH might lead to the same pulmonary changes as CH [4], but CIH exposure associated with intermittent labor at high altitude has not been studied as thoroughly. A prevalence of pulmonary arterial hypertension up to 4% has been reported among subjects exposed to long-term CIH [5]. Therefore, attempting to understand the complexity of the molecular mechanisms involved in long-term CIH-related pulmonary hemodynamic changes may lead to clarifications in the pathophysiology underlying this and other forms of hypoxia-associated pulmonary diseases.

One of the molecules that plays a key role in regulating vasomotor function under hypoxic conditions is nitric oxide (NO) [6]. NO, which is derived from endothelial cells, dilates almost all types of vessels by stimulating soluble guanylyl cyclase (sGC), resulting in increased cyclic GMP in smooth muscle cells [7]. Asymmetric dimethylarginine (ADMA) is a competitive nitric oxide synthase (NOS) inhibitor that has been identified as a regulator of NO production in vivo [5]. ADMA is formed by the dimethylation of L-arginine residues by arginine methyltransferases and is released by subsequent proteolysis [8]. Leone et al. [9] first reported that endogenous ADMA inhibits endothelium-dependent vasodilation in vitro. Almost 80% of ADMA is degraded by a group of hydrolases called dimethylarginine dimethylaminohydrolases (DDAHs) [8]. Two subtypes of DDAHs, DDAH1 and DDAH2, are known and differ in their tissue distribution and their capacity to degrade ADMA. Currently, DDAH is under investigation as a novel therapeutic target to directly regulate ADMA concentrations and indirectly regulate NO [10].

It is generally accepted that hypoxia is associated with a high burden of oxidative stress. In addition to the many interactions between the L-arginine/NO pathway and oxidative stress, interactions also exist between the ADMA/DDAH pathway and oxidative stress [11]. However, the role of the ADMA/NO pathway in hypoxia-associated chronic respiratory diseases has remained controversial [12]. We recently observed a dramatic increase in ADMA in plasma from volunteers exposed to CIH and high-altitude dwellers [13].

In order to gain a broader view of the changes in the ADMA/NO pathway during long-term CIH compared with CH, this study aimed to assess the changes in the ADMA/NO pathway, the underlying pulmonary molecular mechanisms involved, and the potential interaction with other molecules, such as ROS, in lung tissue as a possible explanation for hypoxia-induced pulmonary hypertension.

## 2. Methods

### 2.1. Rat Model of CIH

Twenty-four adult Wistar rats (3 months old) were used for the experiments. The rats were randomly assigned to three groups: CIH2x2 (*n* = 8; 2 days of hypobaric hypoxia/2 days of normoxia), CH (*n* = 8; sustained hypoxia), and NX (*n* = 8; permanent normoxia, for 30 days; this rat model of long-term CIH exposure has been previously used in studies of high-altitude changes [14, 15]). To simulate geographical high altitude, the rats were kept in a hypobaric chamber at 428 Torr, which is equivalent to an altitude of 4,600 m, at Universidad Arturo Prat facilities. After 30 days of exposure, the rats were anesthetized with 0.8 mg ketamine/kg and were subjected to a fatal thoracotomy under anesthesia. Lung tissue was snap-frozen for mRNA and protein extraction as well as measurement of L-arginine, ADMA, and enzyme activities. All the measurements, with the exception of physiological parameters, were performed in lung tissue.

This protocol was approved by the Ethics Committee of Universidad Arturo Prat, and the animals were handled according to international protocols and under veterinary care.

### 2.2. Physiological Measurements

The extent of right ventricular hypertrophy (RVH) was estimated by calculation of the ratio of the right ventricle over the left ventricle plus the septum (RV/LV + S) according to [16]. Weight was measured using an Acculab V-1200® (Chicago, IL, USA) electronic balance 1 h after the chamber descended. For blood pressure and heart rate measurements, an inflatable tail-cuff and a pressure sensor (RTBP1003-220; Kent Scientific®, Torrington, CT, USA) were used following a previously validated method [17]. The data were collected and analyzed using the RTBP-001-DS worksheet (Kent Scientific, Torrington, CT, USA). The hematocrit was measured from blood collected by tail vein puncture at baseline and after 30 days of intervention. An Eppendorf AG® (Hamburg, Germany) microcentrifuge was used to measure the hematocrit level, and the hemoglobin level was measured using a Coulter Electronics Counter, Cell Dyn 3700®, and Abbott® (Santa Clara, CA, USA).

### 2.3. Measurement of L-Arginine and ADMA

Mass spectrometric determination of L-arginine and ADMA in the lung tissue was performed as previously described using a fully validated high-throughput liquid chromatography/tandem mass spectrometry (LC-MS/MS) assay [18, 19]. In brief, samples were analyzed using 96-well 0.20 *μ*m microfiltration plates precoated with 40 pmol of [^2^H_6_]-ADMA and 800 pmol of L-[^2^H_7_]-L-arginine (internal standards). After conversion to their butyl ester derivatives, the analytes were evaluated on a Varian 1200L Triple Quadrupole MS® (Varian, Walnut Creek, CA, USA) in the positive electrospray ionization (ESI+) mode. The L-arginine/ADMA ratio, which is considered a strong indicator of NOS substrate availability, was calculated [20].

### 2.4. DDAH Activity

The DDAH activity in the lung tissue was determined as previously described [21]. In brief, tissue samples were homogenized in PBS with protease inhibitors to minimize possible interference from endogenously formed ADMA. The homogenate was centrifuged in a precooled (4°C) centrifuge for 5 min at 12,000 ×g. Fifty-microliter aliquots of the resulting supernatant were added to 50 *μ*L aliquots of PBS containing 20 *μ*M [^2^H_6_]-ADMA and were incubated at 37°C for 60 min. The reactions were stopped, and [^2^H_6_]-ADMA was determined by LC-MS/MS as described above.

### 2.5. Arginase Activity

Arginase activity was determined using urea measurement by the conversion of L-arginine to urea. A lung tissue sample (30 mg) was homogenized using RIPA buffer (Homogenizer Glas-Col®, Terre Haute, IN, USA) and was then centrifuged at 4,000 rpm for 2 min over ice at 4°C. To activate arginase, the tissue was heated at 55°C (Tembloc PSELECTA®, Unitronic, Orlando, FL, USA) with the addition of 75 *μ*L of buffer (50 mM Tris-HCl with 10 mM MnCl_2_) for 10 min. L-Arginine hydrolysis was accomplished by incubation with 50 *μ*L of L-arginine (0.5 M, pH 9.7) at 37°C for 1 h. To stop the reaction, 400 *μ*L of acid mixture (H_2_SO_4_ : H_3_PO_4_ : H_2_O, 1 : 3 : 7) was added. Urea determination was performed by spectrophotometry (Spectronic GENESYS 6®, Madison, WI, USA) at 540 nm, after the addition of 25 *μ*L of *α*-isonitrosopropiophenone (9% absolute ethanol) followed by heating at 100°C for 45 min.

### 2.6. Measurement of Thiobarbituric Acid Reactive Substances/Malondialdehyde

Lung tissue (50 mg) was homogenized (Homogenizer Glas-Col, Terre Haute, IN, USA) at 4,000 rpm for 2 min with RIPA buffer over ice. One hundred microliters of homogenized tissue was aliquoted into a sterile tube combined with 200 *μ*L of trichloroacetic acid (TCA) reagent and mixed thoroughly. After mixing, the sample was centrifuged at 2,200 ×g for 15 min at 4°C. The absorbance of the supernatant was measured at 532 nm. After adding 200 *μ*L of thiobarbituric acid (TBA, 0.67%), the sample was heated to 100°C for 30 min. The malondialdehyde (MDA) concentration was calculated by spectrophotometry (532 nm) (Spectronic GENESYS 6, Madison, WI, USA).

### 2.7. Real-Time PCR (qRT-PCR)

For qRT-PCR analysis, total RNA was isolated by homogenization of lung tissue in RNAzol reagent (Fa. Biozym, Hessisch Oldendorf, Germany). Reverse transcription was performed using a RevertAid H Minus Strand cDNA Synthesis Kit (Thermo Scientific®, Waltham, MA, USA) according to the manufacturer's recommendations. qRT-PCR was performed on a TaqMan 9700HT system (Roche®, Indianapolis, IN, USA) and an Agilent Stratagene Mx3000P Real-Time PCR system (Santa Clara, CA, USA) using Maxima® SYBR Green/ROX qPCR Master Mix (Thermo Scientific, Waltham, MA, USA). The sequences of the primers for the rat oligonucleotides were designed based on the NCBI GenBank. The qPCR primer sequences are presented in Additional File 1 in Supplementary Material available online at http://dx.doi.org/10.1155/2016/6578578.

### 2.8. Western Blot

Western blot analysis was performed according to a standard protocol using monoclonal antibodies against vasodilator-stimulated phosphoprotein (VASP). Phosphorylated VASP which is the active protein (pVASP, Ser239) and nonphosphorylated VASP (Cell Signaling Technology®, Danvers, MA, USA; #3112 and #3114) were measured, and the pVASP/VASP ratio was calculated as an indirect measurement of sGC-dependent NO activity. Additionally, the levels of dimethylarginine dimethylaminohydrolases (DDAH1 and DDAH2) were determined. As an internal expression control, eukaryotic initiation factor 4E (elF4E) C46H6 (Cell Signaling Technology, Danvers, MA, USA; #2067) was used [22]. In brief, lung tissue was homogenized in lysis buffer containing sodium dodecyl sulfate and a protease inhibitor cocktail (Complete Ultra Tablets, Roche Diagnostics®, Indianapolis, IN, USA). Extracts containing equal amounts of protein were denatured and separated on 10% (SDS-PAGE) polyacrylamide gels. After separation, the proteins were transferred to a nitrocellulose membrane (Protran BA85; Whatman®, GE Healthcare, Fairfield, CT, USA) and were incubated with the corresponding primary antibody and later with the appropriate horseradish peroxidase- (HRP-) conjugated secondary antibodies. The protein fragments were visualized using an enhanced chemiluminescence (ECL) detection kit (Pierce®, ECL Western Blotting Substrate; Thermo Fisher Scientific®, Rockford, IL, USA). Relative quantification was performed using densitometry techniques (GeneSnap®; SynGene, Synoptics, Cambridge, UK).

### 2.9. Data Analysis

The results were analyzed using SPSS 17.0 (SPSS, Inc.®, Chicago, IL, USA). Means, standard deviations, standard errors, and confidence intervals were calculated for each parameter. Normality was established using the Kolmogorov-Smirnov test, and all data were normally distributed. Statistical analysis of the differences across all testing conditions was performed using one-way analysis of variance (ANOVA) and after obtaining an *F* ratio <0.05. A Bonferroni post hoc test was performed to identify differences between the three groups. Additionally, for differences in time, a repeated-measures ANOVA was performed. For correlation analysis of ADMA and physiological outcomes, one-tailed parametric Pearson correlation was used. Results were considered significant when the *p* value was less than 0.05.

## 3. Results

### 3.1. Rat Physiological Measurements

Both hypoxic groups lost weight compared with the NX group (*p* < 0.01), and the weight loss was greater in the CH rats (Table 1). Residual food was left only in the first days of the experiment. As expected, the hematocrit increased in the hypoxic groups (*p* < 0.01) and was greater in the CH rats (*p* < 0.01) (Table 1). The systolic blood pressure increased significantly in the CH group (171.9 ± 1.1 versus 176.7 ± 4.6 mmHg; *p* < 0.01), whereas this value remained stable in the NX (171.1 ± 4.7 versus 171.4 ± 3.0 mmHg) and CIH2x2 (171.8 ± 5.3 versus 172.4 ± 5.6 mmHg) groups. Significant changes in the heart rate were not detected in any of the groups.

### 3.2. Right Ventricle Hypertrophy Ratio (RVH)

**Figure 1:**
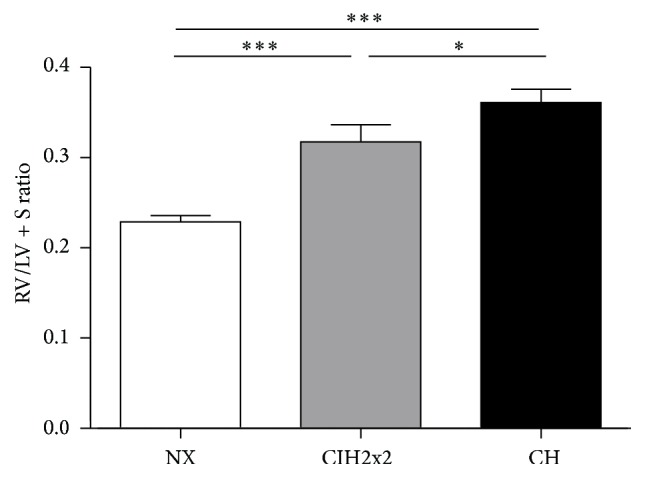
Right ventricle hypertrophy weight (g) ratio (RV/LV + S (right ventricular/left ventricular + septum)) of rats exposed to normoxia (NX), chronic intermittent hypoxia (CIH2x2), or chronic hypoxia (CH). Values are expressed as means and standard errors (SE). ^*∗*^ *p* < 0.05; ^*∗∗∗*^ *p* < 0.001; *n* = 8 in each group.

Both CH and CIH2x2 exhibited an RVH, which was expressed as the RV/LV + S ratio. Rats exposed to CIH2x2 showed a slightly lower ratio than CH rats (*p* < 0.05; Figure 1).

### 3.3. Formation and sGC-Dependent Activity of NO

**Figure 2:**
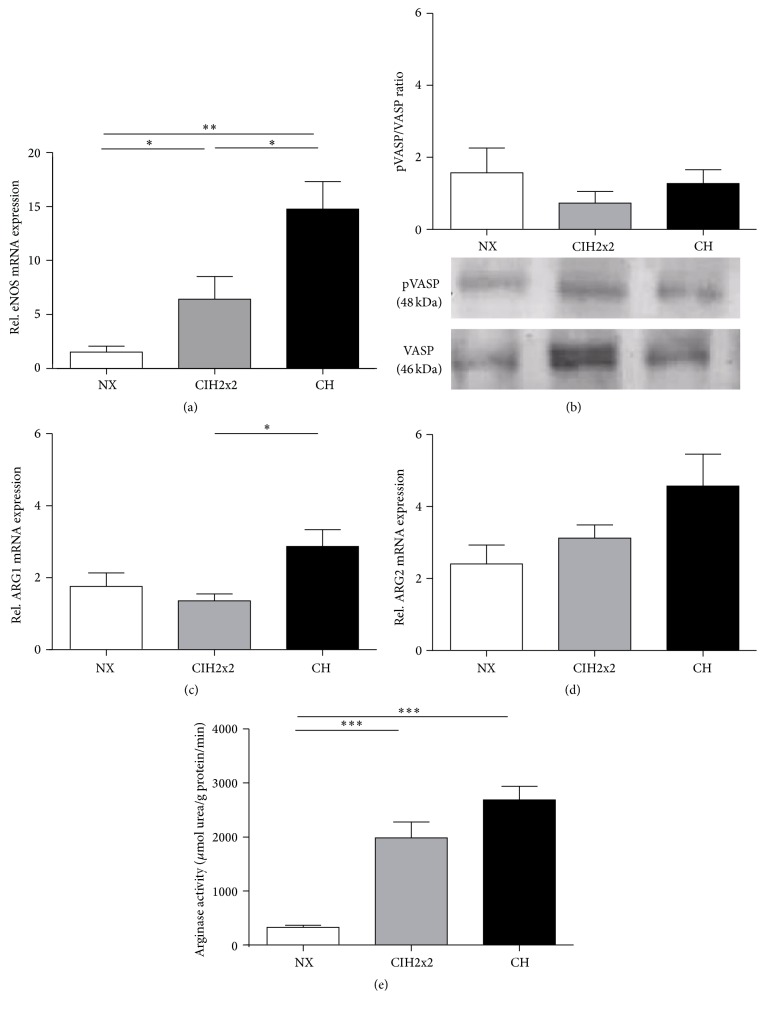
Comparison of (a) mRNA expression of eNOS by qRT-PCR and (b) sGC-dependent bioavailability of NO, expressed as the pVASP/VASP protein ratio. Representative bands on western blots are presented. The substrate availability of NOS, expressed as (c) mRNA expression of arginase isoform I determined by qRT-PCR, (d) mRNA expression of arginase isoform II determined by qRT-PCR, and (e) arginase activity (*μ*mol urea/g protein/min) determined by spectrophotometry in the lungs of rats exposed to normoxia (NX), chronic intermittent hypoxia (CIH2x2), or chronic hypoxia (CH). Values are expressed as means and SE. ^*∗*^ *p* < 0.05; ^*∗∗*^ *p* < 0.01; ^*∗∗∗*^ *p* < 0.001; *n* = 8 in each group.

**Figure 3:**
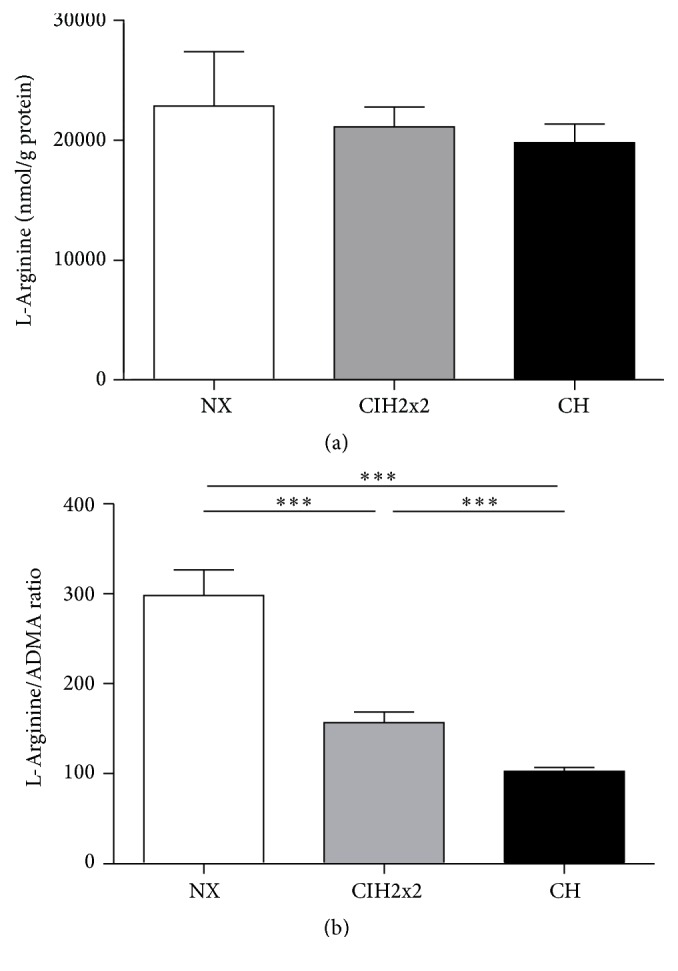
Comparison of (a) L-arginine levels (nmol/g protein) by mass spectrometry and (b) the L-arginine/ADMA ratio in the lungs of rats exposed to normoxia (NX), chronic intermittent hypoxia (CIH2x2), or chronic hypoxia (CH). Values are expressed as means and SE. ^*∗∗∗*^ *p* < 0.001; *n* = 8 in each group.

Despite a progressive increase (up to four-fold in CIH2x2 and eight-fold in CH) in endothelial NOS (eNOS) mRNA expression (*p* < 0.001; Figure 2(a)) in the lung tissue, the ratio of pVASP to total VASP was unchanged in both hypoxic groups (*p* NS; Figure 2(b)). The NOS substrate availability, which relates to the mRNA expression and activity of arginases, the enzymes responsible for degrading L-arginine, was also affected by hypoxia. The relative ARG1 mRNA expression level in lung tissue was increased during CH (*p* < 0.05; Figure 2(c)), whereas ARG2 exhibited a nonsignificant increasing trend for rats under both forms of hypoxia (Figure 2(d)). Although there were only marginal differences in mRNA expression, the arginase activity was strikingly increased during hypoxia (*p* < 0.001), without differences between CIH2x2 and CH (Figure 2(e)). However, the L-arginine tissue concentration in the lung was not affected by hypoxia (Figure 3(a)), whereas the L-arginine/ADMA ratio decreased according to the level of hypoxia (*p* < 0.001; Figure 3(b)).

### 3.4. ADMA Formation and Degradation

**Figure 4:**
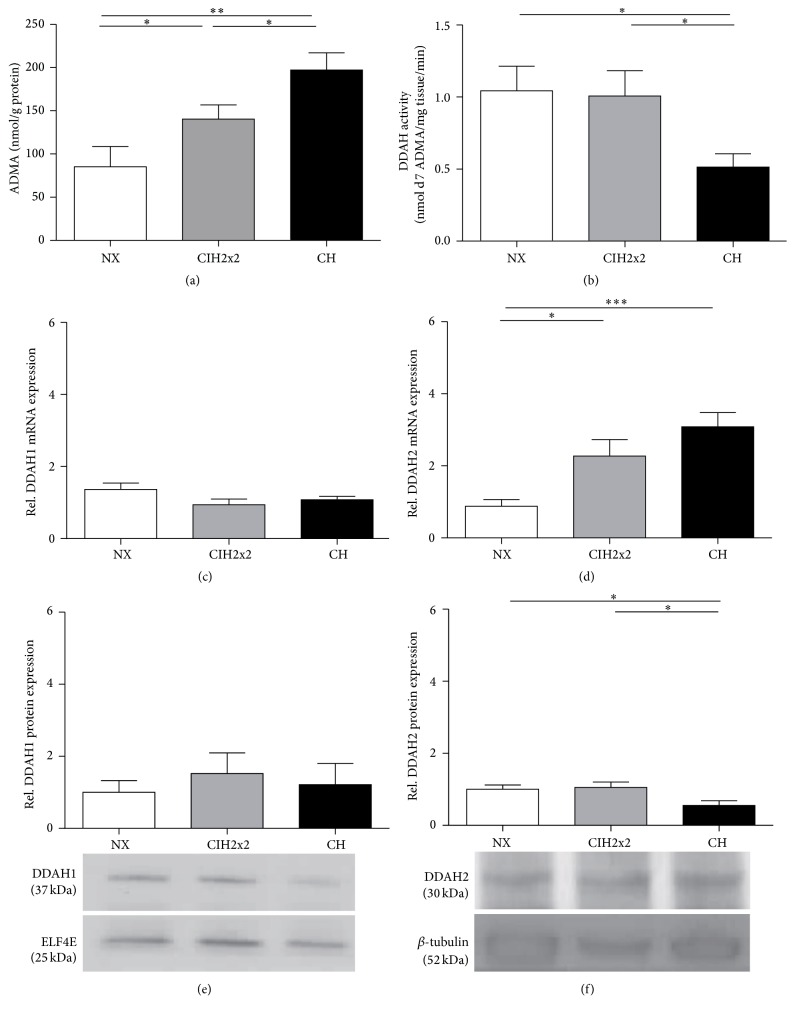
Comparison of (a) ADMA concentrations (nmol/g protein) determined by LC-MS/MS assays, (b) DDAH enzyme activity (nmol d7 ADMA/mg tissue/min) determined by LC-MS/MS assays, (c) mRNA expression of DDAH1 determined by qRT-PCR, (d) mRNA expression of DDAH2 determined by qRT-PCR, (e) DDAH1 protein expression levels determined by western blot, and (f) DDAH2 protein expression levels determined by western blot. Respective representative bands on western blots of lung tissue from rats exposed to normoxia (NX), chronic intermittent hypoxia (CIH2x2), or chronic hypoxia (CH) are presented. Values are expressed as means and SE. ^*∗*^ *p* < 0.05; ^*∗∗*^ *p* < 0.01; ^*∗∗∗*^ *p* < 0.001; *n* = 8 in each group.

Clearly, hypoxia increased the ADMA tissue concentrations in the lung (CIH2x2 *p* < 0.05; CH *p* < 0.05, but CIH2x2 produced intermediate values Figure 4(a)). The DDAH lung tissue activity was reduced only in CH rats (*p* < 0.05; Figure 4(b)). The relative DDAH1 mRNA and protein levels did not change in either hypoxic group (*p* NS; Figures 4(c) and 4(e)). Conversely, DDAH2 mRNA expression significantly increased (*p* < 0.001), whereas DDAH2 protein expression slightly decreased in the CH group (*p* < 0.05; Figures 4(d) and 4(f)). This latter finding correlates with the DDAH activity result.

### 3.5. Correlation of ADMA and Physiological Outcome

After 30 days of chronic hypoxia ADMA was positively correlated with systolic blood pressure (Table 2), whereas in the status of CIH no correlation was seen. In contrast to that, the heart rate was negatively correlated to ADMA after 30 days of CIH whereas for CH no correlation was seen (Table 2).

### 3.6. NADPH Activity and Oxidative Stress under Hypoxia

**Figure 5:**
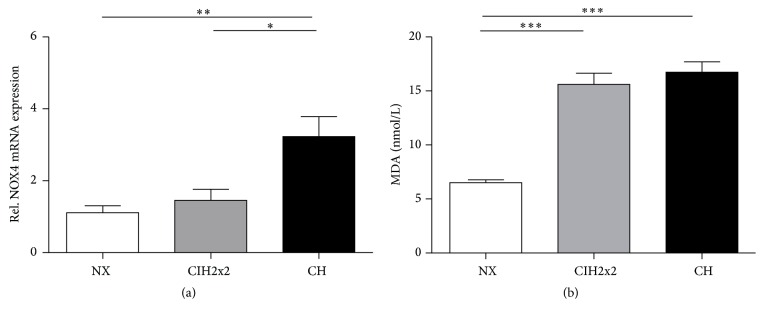
Comparison of (a) mRNA expression levels of NOX4 determined by qRT-PCR and (b) MDA concentrations (nmol/L) determined by spectrophotometry of lung tissue from rats exposed to normoxia (NX), chronic intermittent hypoxia (CIH2x2), or chronic sustained hypoxia (CH). Values are expressed as means and SE. ^*∗*^ *p* < 0.05; ^*∗∗*^ *p* < 0.01; ^*∗∗∗*^ *p* < 0.001; *n* = 8 in each group.

CH increased NADPH subunit NOX4 mRNA expression (*p* < 0.01), whereas in CIH2x2, no difference from normoxia was observed (Figure 5(a)). The MDA concentration within the lung increased up to 2-fold, under both hypoxic conditions (*p* < 0.001) without significant differences between them (*p* NS) (Figure 5(b)).

## 4. Discussion

Our study has seven major findings: (1) Under both hypoxic conditions, rats developed moderate RVH. (2) eNOS expression increased in long-term CIH2x2 and CH, but the activation of sGC by NO did not. (3) The L-arginine concentration was unaffected despite the fact that arginase activity was enhanced under both hypoxic conditions. (4) ADMA concentrations in the lung increased in the long-term CIH2x2 and CH due to a hypoxia-induced reduction of DDAH activity in CH. (5) ADMA concentrations were positively correlated with systolic blood pressure in long-term CH. (6) Both hypoxic conditions led to a strong increase in biomarkers for oxidative stress. (7) Both CIH and CH exhibited similar effects, but they were lower or absent in the CIH group.

Hypoxic pulmonary vasoconstriction (HPV) occurs in response to hypoxia [23]. If this exposure is sustained, a subsequent vascular remodeling of pulmonary arteries develops, causing an increase in pulmonary vascular resistance and leading to RVH as part of hypoxia-induced pulmonary hypertension, resulting in premature death [24–27]. Consistent with these studies, we observed RVH in both hypoxic groups, although RVH was more moderate in CIH2x2, as previously described [14]. The molecular mechanisms involve components both upstream of the endothelial NO pathway and mechanisms leading to oxidative stress downstream in the smooth muscle cells [28, 29].

Therefore, vascular response to hypoxia is critical, and the endothelium plays a crucial role. Specifically, the endothelium is one major source of the vasodilator NO.

Our current study shows that eNOS mRNA expression is enhanced in hypoxia as expected. However, the increased mRNA expression of eNOS did not result in an elevation of NO-activated sGC-dependent activity as expressed as the pVASP/VASP ratio (an indirect measurement of NO-activated sGC-dependent activity) [30, 31]. There are several possible explanations for this result.

First, a reduction in DDAH activity in the CH rat lung was observed, which resulted in an increased ADMA tissue concentration. Interestingly, the reduction in DDAH activity appears to be dependent only on DDAH2. Although we have recently demonstrated that DDAH1 is the isoform genetically associated with ADMA [32], the influence of DDAH2 on ADMA appears to increase in diseased states, which is consistent with our results. Subsequently, as ADMA is known to displace L-arginine (NOS substrate) from the binding site of NOS and thereby leads to competitive NOS inhibition, lower NO substrate availability could occur. This possibility is supported by a hypoxia-induced decrease detected in the L-arginine/ADMA ratio, an indicator of NOS substrate availability [20] and the correlation of ADMA and systolic blood pressure pointing to a systemic endothelial dysfunction.

Second, a hypoxia-induced increase in oxidative stress occurred, as evidenced by the enhanced NOX4 mRNA expression. NOX4 is mainly expressed in vascular smooth muscle cells (VSMCs), and NADPH oxidase activity in VSMCs is largely dependent on NOX4 mRNA expression [33]. Additionally, under hypoxic conditions, superoxide radicals are produced, enhancing lipid peroxidation. It is known that superoxide radical-mediated NO degradation attenuates its capacity to stimulate cGMP and the potential beneficial actions of NO on pulmonary arterial vasodilation [34].

Accordingly, in this study, both CIH and CH led to a strikingly increased MDA level along with increased NOX4 mRNA expression only in CH rats, suggesting a burst of superoxide radicals. These results are coincident with a diminished NO bioavailability and increased superoxide anions in rat pulmonary arteries observed by confocal microscopy [35]. Moreover, MDA might also inhibit DDAH activity, as previously demonstrated for 4-HNE (4-hydroxy-2-nonenal) [36]. This effect could also explain why our results indicate reduced DDAH activity.

L-Arginine is converted into L-ornithine by arginases, thereby diminishing its availability as a NOS substrate. Arginases have recently been reported to play important roles in the pathogenesis of various pulmonary disorders [37]. A steep increase in lung arginase activity is clearly demonstrated in our results, and this increase was not followed by an equal decrease in L-arginine. Interestingly, only arginase isoform I gene expression was significantly increased, with an increasing trend in arginase isoform II expression. This latter is not coincident with a previous report showing an increase in arginase II isoform in CIH, but using different methodology and measurement techniques [38]. Therefore, given the L-arginine/ADMA ratio results, we could postulate that although the L-arginine concentration exhibits almost no changes, the bioavailability of this substrate is imbalanced or impaired.

Our study has some limitations. We used RVH, as a generally accepted indirect marker of increased pressure within the pulmonary circulation. Due to the increases in NOX4 mRNA expression and MDA formation, we inferred a rise in ROS, but we did not measure ROS directly. Instead of measuring NO directly, we used an established indirect measurement by determining the pVASP/VASP ratio. Furthermore, all measurements were made in whole-lung homogenates, which may not represent what specifically occurs in the pulmonary endothelium. Nevertheless, most of our results are consistent with those that have been separately described, whereas we have attempted to describe a wider and more integrated picture of these changes in the lung under long-term CIH exposure.

## 5. Conclusions

This observational study demonstrated that long-term CIH and CH in rats led to an imbalance in the NO pathway, where ADMA and oxidative stress could be responsible for impaired pulmonary vascular function by inhibiting or impairing signal transduction (endothelial dysfunction). Both hypoxic conditions resulted in similar effects on NO signaling in the lungs, but CIH had a more limited or no effect on certain parameters. The clinical relevance of ADMA and DDAH and their roles in the development of hypoxic pulmonary hypertension in CIH remain to be determined, opening a new field of research as biomarkers or drug targets in either HAPH or other pulmonary hypoxic conditions.

## Supplementary Material

The Supplementary Material shows primer sequences used for mRNA expression analysis by qRT-PCR in whole-lung homogenates of rats exposed to chronic intermittent hypoxia, chronic hypoxia and normoxia. The sequence of the primers for rats oligonucleotide were designed based on NCBI GenBank.

## Figures and Tables

**Table 1:** Weight and hematocrit in rats exposed to normoxia (NX), chronic intermittent hypoxia (CIH2x2), and chronic hypoxia (CH) at day 0 (baseline) and day 30 (after exposure). Data are expressed as means ± standard error (SE), and differences were obtained by repeated measures ANOVA; ^*∗*^ *p* < 0.01; ^*∗∗*^ *p* < 0.001 compared with the baseline.

	NX		CIH2x2		CH	
	Baseline	After exposure	Baseline	After exposure	Baseline	After exposure
Weight (g)	229.5 ± 19.7	226.0 ± 14.2	226.3 ± 25.1	191.0 ± 17.4^**∗**^	228.7 ± 33.9	178.2 ± 20.2^**∗∗**^
Hematocrit (%)	40.3 ± 1.6	42.4 ± 2.0	39.2 ± 2.5	53.7 ± 3.7^**∗**^	39.2 ± 3.0	63.5 ± 4.2^**∗**^

**Table 2:** Correlation of ADMA, systolic blood pressure (SBP), and heart rate (HR) of rats exposed 30 days to chronic intermittent hypoxia (CIH2x2) and chronic hypoxia (CH); *p* = *p* value; *R* = Pearson correlation coefficient; ^*∗*^one-tailed significance level = 0.05.

	SBP CIH2x2		SBP CH		HR CIH2x2		HR CH	
	*R*	*p*	*R*	*p*	*R*	*p*	*R*	*p*
ADMA	−0.041	0.462	0.635	0.045^**∗**^	−0.633	0.046^**∗**^	−0.277	0.253
