# Supplementary material for: Long-Term Chronic Intermittent Hypobaric Hypoxia in Rats Causes an Imbalance in the Asymmetric Dimethylarginine/Nitric Oxide Pathway and ROS Activity: A Possible Synergistic Mechanism for Altitude Pulmonary Hypertension?
Source: Pulm Med. 2016 May 30;2016:6578578. doi: 10.1155/2016/6578578 (PMC4904121; doi:10.1155/2016/6578578)
Supplement: Supplementary file 1 — The Supplementary Material shows primer sequences used for mRNA expression analysis by qRT-PCR in whole-lung homogenates of rats exposed to chronic intermittent hypoxia, chronic hypoxia and normoxia. The sequence of the primers for rats oligonucleotide were designed based on NCBI GenBank. [file 6578578.f1.pdf]

Additional File 1: Primer sequences used for mRNA expression analysis by qRT-PCR in whole-lung homogenates of rats exposed to chronic intermittent hypoxia, chronic hypoxia and normoxia. The sequence of the primers for rats oligonucleotide were designed based on NCBI GenBank .

**rArgI_for**	5´-TTGTCAGCGGAGTGTTGATGTC-3´
**rArgI_rev**	5´-CAGAGACCCAGAAGAATGGAAC -3´
**rArgII_for**	5´-AAGAAAGGAGTGGAATATGGCCCAGC-3´
**rArgII_rev**	5´-TAGCCACCTGACACAGCTCTACTAA-3´
**reNos_for**	5´-TGATCCTAACTTGCCTTGCATCCT-3´
**reNos_rev**	5´-ACTCCACGCTGCTGGGCGTC-3´
**Nox4 for**	5´- TGCCGCACAGTCCTGGCTTACC -3´
**Nox4 rev**	5´- GGGCGTTCACCAAGTGGGCAGC -3´
**rHprt_for**	5´-TCCTCATGGACTGATTATGGACA-3´
**rHprt_rev**	5´-TAATCCAGCAGGTCAGCAAAGA-3´
**rDDAH1_for**	5´-GAGGCTTTGGAAAAACTTCAGCTCA-3´
**rDDAH1_rev**	5´-GCCAAGATCTCAGCACCTCGTTGAT-3´
**rDDAH2_for**	5´-GCATCTGCGCGGCCTCTGTG-3´
**rDDAH2_rev**	5´-AGTCACTCGCTGCGTCATCTGGGAG-3´
